# Long Non-Coding RNAs (lncRNAs) Dysregulated in Tumor Progression to Castration-Resistant Prostate Cancer

**DOI:** 10.1200/GO.22.66000

**Published:** 2022-05-05

**Authors:** Sabrina Ledesma-Bazan, Elba Vazquez, Geraldine Gueron, Javier Cotignola

**Affiliations:** ^1^ IQUIBICEN (CONICET-UBA), Buenos Aires, Argentina

## Abstract

The dysregulation of non-coding RNAs is implicated in the development and progression of different pathological processes, including cancer. Prostate cancer is known for having a wide spectrum of clinical outcomes, ranging from an indolent tumor to a lethal metastatic castration resistant cancer (CRPC). However, the biology of the processes leading to these phenotypes remains elusive. The aim of this study was to identify lncRNAs involved in the progression of prostate tumors to CRPC.

**METHODS:**

We browed public repositories and downloaded raw RNAseq data from primary prostate adenocarcinomas (pre- and post-androgen deprivation therapy (ADT); n = 40 and n = 11 (paired to 11 pre-ADT), respectively) and CRPC (n = 8). We performed differential gene expression of 17,009 lncRNAs with R/Bioconductor using two statistical models: DESeq2 and edgeR.

**RESULTS:**

We identified 12 lncRNAs that responded to ADT and were further dysregulated in CRPC; three of them were previously reported as players in prostate cancer development and progression (PCA3, PCAT18 and PCGEM1), validating the pipeline used in this study. Interestingly, all of them showed the same expression pattern: (1) post-ADT tumors had significantly lower expression compared with pre-ADT tumors; and (2) CRPCs showed significant higher levels compared with post-ADT tumors, and the expression was similar, or even higher, to primary pre-ADT tumors. Unsupervised clustering analysis revealed that the expression profile of these 12 lncRNAs could cluster post-ADT and CRPC samples apart. Primary tumors pre-ADT were more heterogeneous. This suggests that primary prostate tumors which might potentially progress to CRPC might be detected at the time of diagnosis according to their lncRNA expression profile.

**CONCLUSION:**

Herein we describe a gene expression profile comprised of 12 lncRNAs that respond to hormone therapy and that are dysregulated in CRPC. The study of this expression signature might help to characterize primary tumors with low/high risk of progression to CRPC.

